# Cutaneous Adnexal Tumours in an Eastern European Cohort: Clinicopathological Spectrum and Rare Malignant Lesions

**DOI:** 10.3390/cancers18172822

**Published:** 2026-09-01

**Authors:** Andreea Cătălina Tinca, Martin Manole, Raluca-Diana Hagău, Alexandru-Constantin Ioniță, Diana Maria Chiorean, Vicențiu Popa, Adrian-Horațiu Sabău, Sofia Muntean, Iuliu Gabriel Cocuz, Ovidiu Simion Cotoi

**Affiliations:** 1 Department of Pathophysiology, George Emil Palade University of Medicine, Pharmacy, Science and Technology, 540142 Târgu Mureș, Romania; andreea-catalina.tinca@umfst.ro (A.C.T.); diana.chiorean@umfst.ro (D.M.C.); adrian-horatiu.sabau@umfst.ro (A.-H.S.); sofia.harsan@umfst.ro (S.M.); iuliu.cocuz@umfst.ro (I.G.C.); ovidiu.cotoi@umfst.ro (O.S.C.); 2 Pathology Department, Clinical County Hospital Mureș, 540011 Târgu Mureș, Romania; ralucahagau1995@gmail.com; 3 Military Medicine Institute, 3–5 Institutul Medico-Militar Street, 010919 Bucharest, Romania; alexionita2@gmail.com; 4 Serviciul Județean de Medicină Legală Dâmbovița, 2 Cooperației Street, 130086 Târgoviște, Romania; vicentiu.popa.md@gmail.com

**Keywords:** cutaneous adnexal tumours, sweat gland tumours, hair follicle tumours, histopathology, rare adnexal neoplasms

## Abstract

**Introduction:** Cutaneous adnexal tumours are a heterogeneous group of tumours arising from the adnexal structures of the skin. These include follicular, eccrine, apocrine, and sebaceous lineages. These entities display a wide morphological spectrum with overlapping histopathological features, posing significant diagnostic challenges. This study aims to provide a detailed analysis of these neoplasms. **Materials and Methods:** We conducted a retrospective observational study including patients with primary cutaneous adnexal tumours diagnosed in excisional specimens at our centre between 2018 and 2025. **Results:** A total of 82 primary cutaneous adnexal tumours were analysed in patients aged 24–90 years. A female predominance was observed (62.19%), with a mean age of 56 years among female patients and 60 years among male patients. The head and neck region was the most frequently affected site (70.73%), followed by the upper limb, thorax, and lower limb. Sweat gland tumours markedly outnumbered follicular tumours (79.27% vs. 20.73%), with hidrocystomas and spiradenomas representing the most common benign lesions. Most tumours were benign (91.46%), while atypical spiradenoma (3.66%) and malignant tumours such as eccrine porocarcinoma, trichilemmal carcinoma, and basal cell carcinoma arising in association with trichoblastoma (4.88%) were rare but clinically significant findings. Surgical specimen volumes varied significantly by anatomical site, with scalp and limb lesions reaching the largest dimensions. Margin status differed according to biological behaviour, with malignant tumours showing greater proportion of close or infiltrated margins, while margins were non-assessable in all three atypical cases. Immunohistochemical evaluation supported diagnostic confirmation in atypical and malignant cases. **Conclusions:** Our findings highlight the broad clinical and morphological spectrum of cutaneous adnexal tumours and contribute data from an Eastern European cohort. This study adds regional data to the limited literature on the clinicopathological distribution of cutaneous adnexal tumours and emphasises rare, atypical, and malignant diagnostic scenarios.

## Simple Summary

Cutaneous adnexal tumours are uncommon and diagnostically challenging lesions with diverse morphological patterns. This study analyses 82 primary cutaneous adnexal tumours diagnosed in excisional specimens at a Romanian tertiary pathology department between 2018 and 2025. The study analyses the epidemiological, clinical, topographical and histopathological characteristics of these tumours. These findings provide regional Eastern European data and emphasise the importance of careful histopathological evaluation of these rare lesions.

## 1. Introduction

Cutaneous adnexal neoplasms are a heterogeneous group of neoplasms that include apocrine, eccrine, sebaceous, follicular, and mixed lineages. There is a high variability in age, histological appearance, and epidemiology. Benign tumours are by far the most common and may occur at any age and at any anatomical site. Malignant counterparts are rare and usually correlate with advanced age. These tumours are more frequently seen in men and in White populations. Adnexal neoplasms are important because they can be seen in association with various hereditary syndromes, yet much of their tumourigenesis remains unknown. Important risk factors for the development of these tumours include exposure to ultraviolet light and immunosuppression. Tumours associated with syndromes are multiple, whereas sporadic tumours are usually solitary. Dermoscopy and clinical appearance of the lesions are often non-specific, while immunohistochemistry techniques may also be inconclusive or non-specific. A detailed and extensive histological evaluation remains the most important tool for their diagnosis.

Although cutaneous adnexal tumours (CATs) have been extensively described, most clinicopathological series originate from large Western or Asian centres, while Eastern European data remain limited. In Romania, published cohort-level data on the full histopathological spectrum of cutaneous adnexal tumours are scarce. The present study, therefore, provides regional real-world data from a tertiary pathology centre, focusing not only on tumour frequency and anatomical distribution, but also on the diagnostic challenges posed by atypical and malignant lesions. The novelty of this study lies in documenting the spectrum of adnexal tumours encountered in routine dermatopathology practice in an Eastern European cohort, highlighting the predominance of sweat gland tumours, the high frequency of hidrocystomas, and the rare occurrence of atypical spiradenomas, eccrine porocarcinomas, and basal cell carcinoma most likely arising in association with trichoblastoma.

## 2. Materials and Methods

We performed a retrospective observational study including all cases of primary cutaneous adnexal tumours diagnosed in the Pathology Department of Clinical County Hospital Mureș, a tertiary hospital in Romania, from 2018 to 2025 (*n* = 82).

### 2.1. Histopathological Evaluation and Tumour Classification

Tumours were classified according to the 5th edition of the WHO classification of Skin Tumours and were grouped according to their predominant line of adnexal differentiation.

For sweat gland tumours, classification as eccrine, apocrine, or mixed eccrine-apocrine, or not otherwise specified (NOS) was based primarily on histopathological features. Apocrine differentiation was recognised by characteristic morphological features, particularly an inner layer of columnar cells showing apical cytoplasmic snouting or decapitation secretion. Eccrine differentiation was assigned when morphology was consistent with eccrine ductal differentiation. Lesions showing unequivocal morphological features of both eccrine and apocrine differentiation were classified as mixed eccrine-apocrine.

Hydrocystomas were included among sweat gland tumours in accordance with their conventional eccrine/apocrine classification. Lesions morphologically consistent with hydrocystomas but lacking sufficiently preserved epithelial features in order to establish either an eccrine or apocrine differentiation were classified as NOS hydrocystomas.

Atypical spiradenomas were classified according to the morphological criteria described by Kazakov et al. The diagnosis was based on the overall histopathological appearance rather than mitotic activity alone. These lesions retained the conventional architecture and characteristic cellular components of spiradenoma while showing focal cytopathological atypia, nuclear enlargement, and focally increased mitotic activity.

Histological evaluation was performed using a ZEISS Primostar 3 microscope (Carl Zeiss Microscopy GmbH, Jena, Germany). Mitotic figures were counted across 10 high-power fields (HPFs) using a ×40/0.65 objective and an eyepiece with a field number of 20 each. Each HPF corresponded to an area of approximately 0.196 mm^2^, resulting in a total examined area of approximately 1.96 mm^2^ for 10 HPFs.

### 2.2. Inclusion and Exclusion Criteria

Only primary cutaneous adnexal tumours diagnosed in excisional specimens were included. Incisional biopsies, punch biopsies, curettage specimens, non-adnexal cutaneous tumours, metastatic tumours, and in situ epithelial lesions were excluded. No sebaceous tumours were identified in our database and therefore are not present in our cohort.

During the study period, no primary cutaneous adnexal tumour was identified in our database on an incisional biopsy, punch biopsy, or curettage specimen; therefore, all eligible cases identified for inclusion were excisional specimens.

### 2.3. Volume Analysis

For exploratory purposes, tumour and excision specimen volumes were estimated from three orthogonal dimensions using the ellipsoid approximation. Volumes were expressed in mm^3^ and calculated according to the formula: $V=\frac{\pi }{6}\cdot L\cdot w\cdot h$, where *L*, *w* and *h* represent the maximum length, width, and height (mm), respectively. Measurements were obtained from the macroscopic dimensions recorded in the histopathology reports. This method provides an estimated geometric volume based on the assumption of an approximately ellipsoidal configuration and may therefore be less representative of the true volume of irregularly shaped lesions. Reports missing one or more of the three required dimensions were excluded from the volumetric analysis, and no imputation of missing measurements was performed. Tumour and excision specimen volumes were assessed independently; therefore, cases with complete measurements for one parameter (tumour or specimen) remained eligible for its respective analysis. Specimen volume refers to the volume of the entire surgically excised tissue, encompassing both the tumour and the surrounding non-tumour tissue, whereas tumour volume refers exclusively to the volume of the tumour itself, excluding any adjacent non-tumour tissue.

### 2.4. Tissue Processing Considerations

Tissue specimens were processed using the standard histological techniques employed in our laboratory, comprising formalin fixation and paraffin embedding, followed by haematoxylin–eosin (H&E) staining.

The immunohistochemical reactions were performed on 4 µm-thick sections prepared from formalin-fixed, paraffin-embedded tissue, using an automated immunostainer (Ventana Medical Systems, Inc., Tucson, AZ, USA). All reagents and incubation times were selected according to the manufacturer’s standard protocols. The slides were developed using the OmniMap 3,3′ Diaminobenzidine (DAB) detection kit (Ventana Medical Systems, Inc., Oro Valley, AZ, USA) and were counterstained with haematoxylin.

For further details regarding the immunohistochemical reactions and antibodies used, please refer to Appendix A.

### 2.5. Surgical Margins

Surgical margins were classified into four categories: free, close, infiltrated and non-assessable.

Margins were classified as free when the tumour was located more than 1 mm from the closest assessable surgical margin. A close margin was defined as tumour located within less than 1 mm of the surgical margin without reaching the inked margin. Margins were considered infiltrated when tumour cells reached the inked surgical margin. Margin status was classified as non-assessable when the relationship between the tumour and the margin could not be reliably evaluated.

Where specimen orientation permitted, peripheral and deep margins were evaluated separately according to the available macroscopic and histopathological information. In unoriented specimens, the resection surface was inked and evaluated histologically as the deep surgical margin when no reliable anatomical landmarks allowed further distinction between individual margins.

Specimens in which fragmentation, orientation, or other technical factors prevented reliable evaluation were classified as having non-assessable margins rather than being assigned uncertain margin status.

When multiple margins were assessable, the shortest tumour-to-margin distance was used to determine the degree of distance. Therefore, the closest surgical margin determined the final margin classification used for statistical analysis.

### 2.6. Statistical Analysis

Statistical analysis and data collection, and organisation were performed using the software GraphPad Prism 11 (version 11.0.1) and Microsoft Excel for Microsoft 365 MSO (Version 2606). We assessed data normality of continuous variables (age and volume) using the Shapiro–Wilk test and by analysing Q-Q plots. As appropriate we used parametric (*t*-test and ANOVA) and non-parametric (Mann–Whitney and Kruskal–Wallis tests) statistical tests. According to the data distribution, we described data using the mean ± standard deviation (SD) for variables with a Gaussian distribution and the median with the interquartile range (IQR) for variables with a non-Gaussian distribution. For correlation analysis, we used the non-parametric Spearman correlation test. Statistical tests were not performed for variables with very low frequencies because the resulting estimates would be unreliable, and these instances are explicitly indicated where applicable throughout the Section 3.

For categorical variables, data are presented in the tables as absolute (*n*) and relative (%) frequencies. Statistical tests used included the Chi-square test (standard or with Yates’ correction, as appropriate), and Fisher’s exact test, as required.

All tests were two-tailed, and the level of statistical significance was set at *α* ≤ 0.05, with a 95% confidence interval (CI). For transparency, both significant and non-significant *p* values were reported.

## 3. Results

The Section 3 is divided into two sections. Section 3.1 aims to analyse the epidemiological and pathological characteristics of primary adnexal tumours, while Section 3.2 aims to illustrate the main histopathology and immunohistochemistry features.

### 3.1. Epidemiological and Pathological Characteristics of Primary Adnexal Tumours

#### 3.1.1. Epidemiology and Topography of Primary Cutaneous Adnexal Tumours

A total of 82 primary cutaneous adnexal tumours were included in the study, with an age range of 24–90 years. This section summarises the demographic characteristics of the study population presented in Table 1. Table 2 shows the topographic distribution of adnexal tumours.

#### 3.1.2. Histopathological Analysis of Primary Adnexal Tumours

This subsection characterises the histopathological spectrum of primary adnexal tumours. Table 3 summarises the distribution of tumour diagnoses according to lineage, biological behaviour, and sex. Table 4 and Table 5 evaluate the associations between biological behaviour, tumour lineage and sex, while Table 6 analyses patient age according to clinicopathological characteristics, including biological behaviour, tumour lineage, and anatomical location.

#### 3.1.3. Volumetric Analysis of Primary Adnexal Tumours

This subsection presents the volumetric characteristics of primary adnexal tumours. Table 7 compares both tumour and surgical specimen volumes according to sex, topography and histopathological variables while Table 8 presents correlations between volumetric measurements, patient age and the year of diagnosis.

#### 3.1.4. Surgical Outcome Analysis of Primary Adnexal Tumours

This subsection evaluates surgical outcomes of primary adnexal tumours. Table 9 summarises the distribution of surgical margins in relation to sex, topography and clinicopathological characteristics while Table 10 analyses surgical margins in relation to tumour and specimen volumes and age.

### 3.2. Histopathology and Immunohistochemistry

#### 3.2.1. Benign Tumours

**Figure 1:**
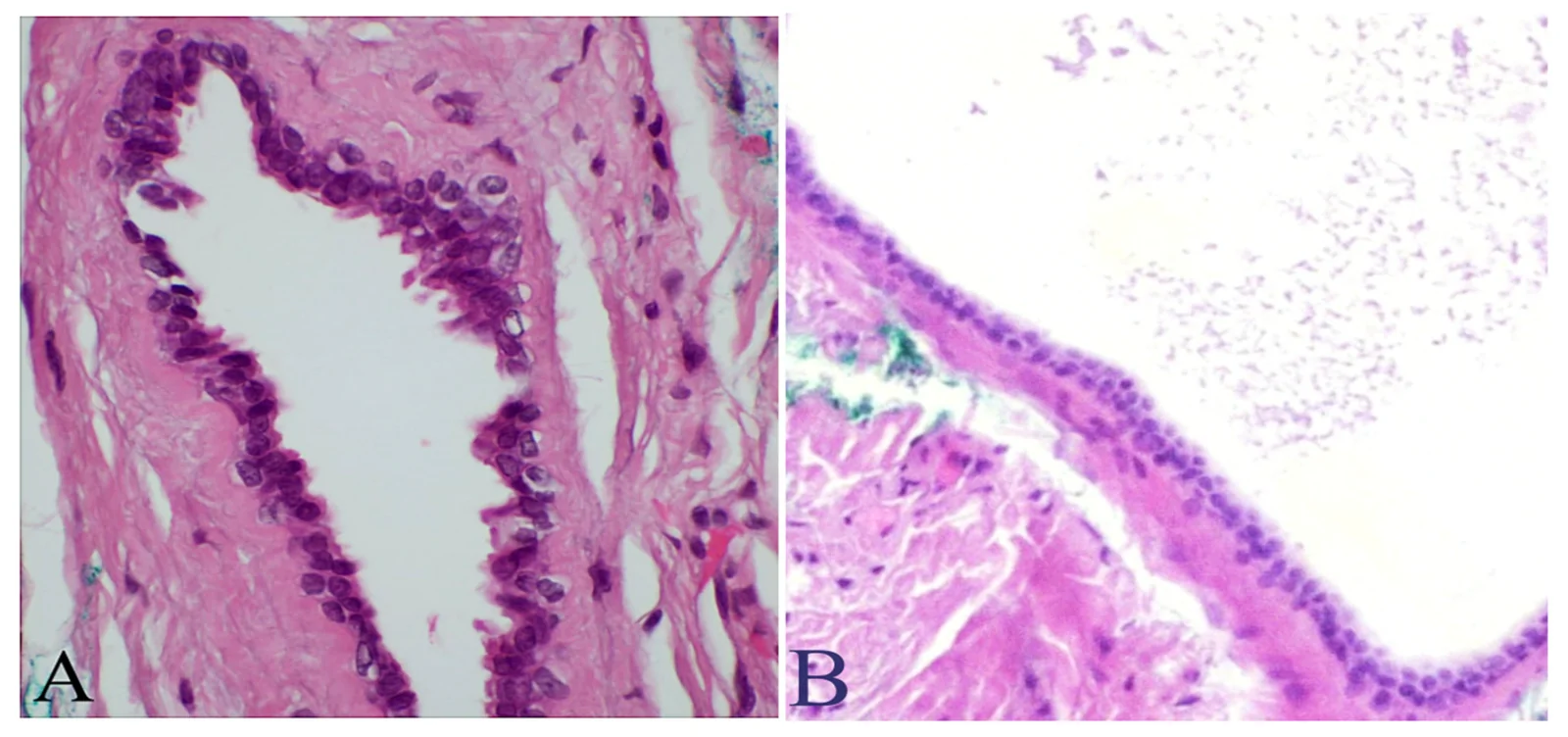
Hidrocystoma. (**A**) Apocrine hidrocystoma, axillae. Cystic lesion lined by double-layered epithelium, with decapitation secretion, HE, 10× objective. (**B**) Eccrine hidrocystoma, eyelid. Cystic lesion lined by a double layer of cuboidal cells. The cells present a small, eosinophilic cytoplasm, and round to ovoid nuclei, without atypia, HE, 10× objective.

**Figure 2:**
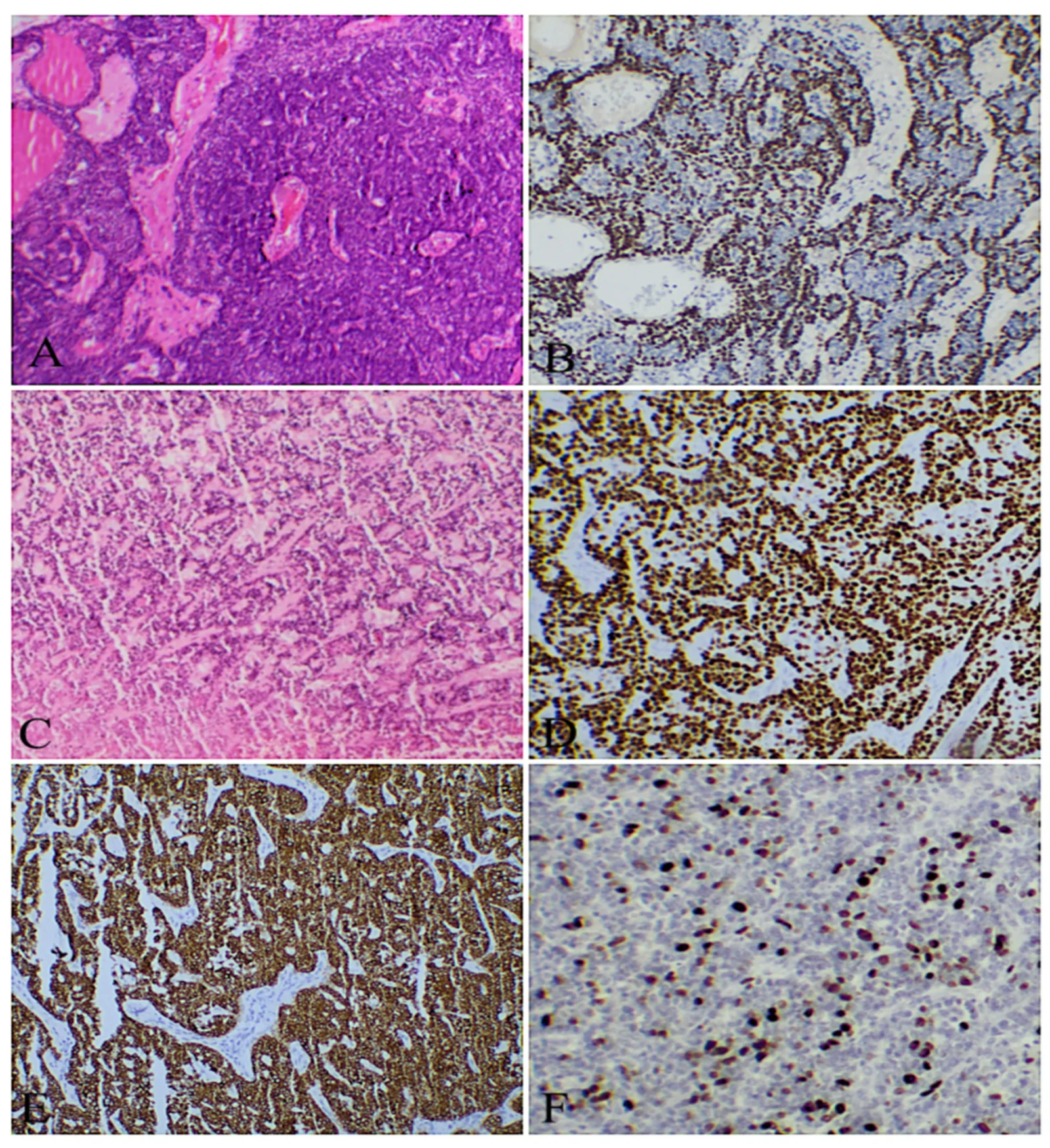
Spiradenoma and atypical spiradenoma. (**A**) Solid proliferation composed of basaloid cells, with cystic changes and well-represented blood vessels, HE, 10× objective. (**B**) Immunohistochemistry with marker p63, 10× objective. (**C**) Atypical spiradenoma, with slight architectural changes and enlarged cells, HE, 10× objective. (**D**) Retained expression of p63 in atypical spiradenoma, 10× objective. (**E**) Expression of marker CK7 in atypical spiradenoma, 10× objective. (**F**) Immunohistochemistry reaction with Ki-67 in atypical spiradenoma, showing overall 15% positivity, 20× objective.

**Figure 3:**
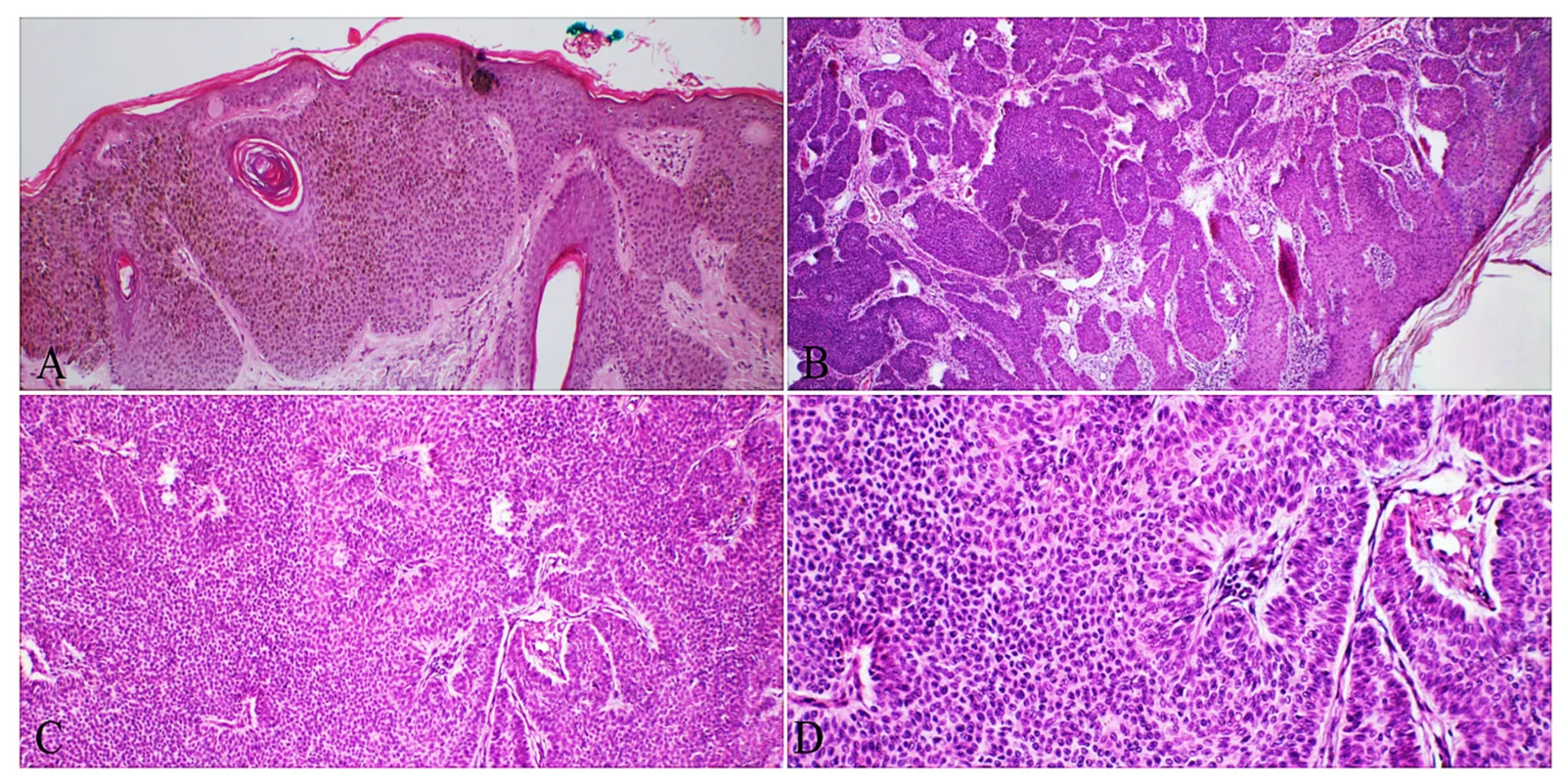
Poroma. (**A**–**D**) Tumour proliferation with solid architecture, composed of uniform, monomorphic cells. (**A**) Melanin pigment can be observed, HE, 5× objective. (**B**) Anastomosing cords are seen towards the depth of the dermis, HE, 5× objective. (**C**) Cytological details showing bland cells with uniform, round nuclei, HE, 10× objective.

**Figure 4:**
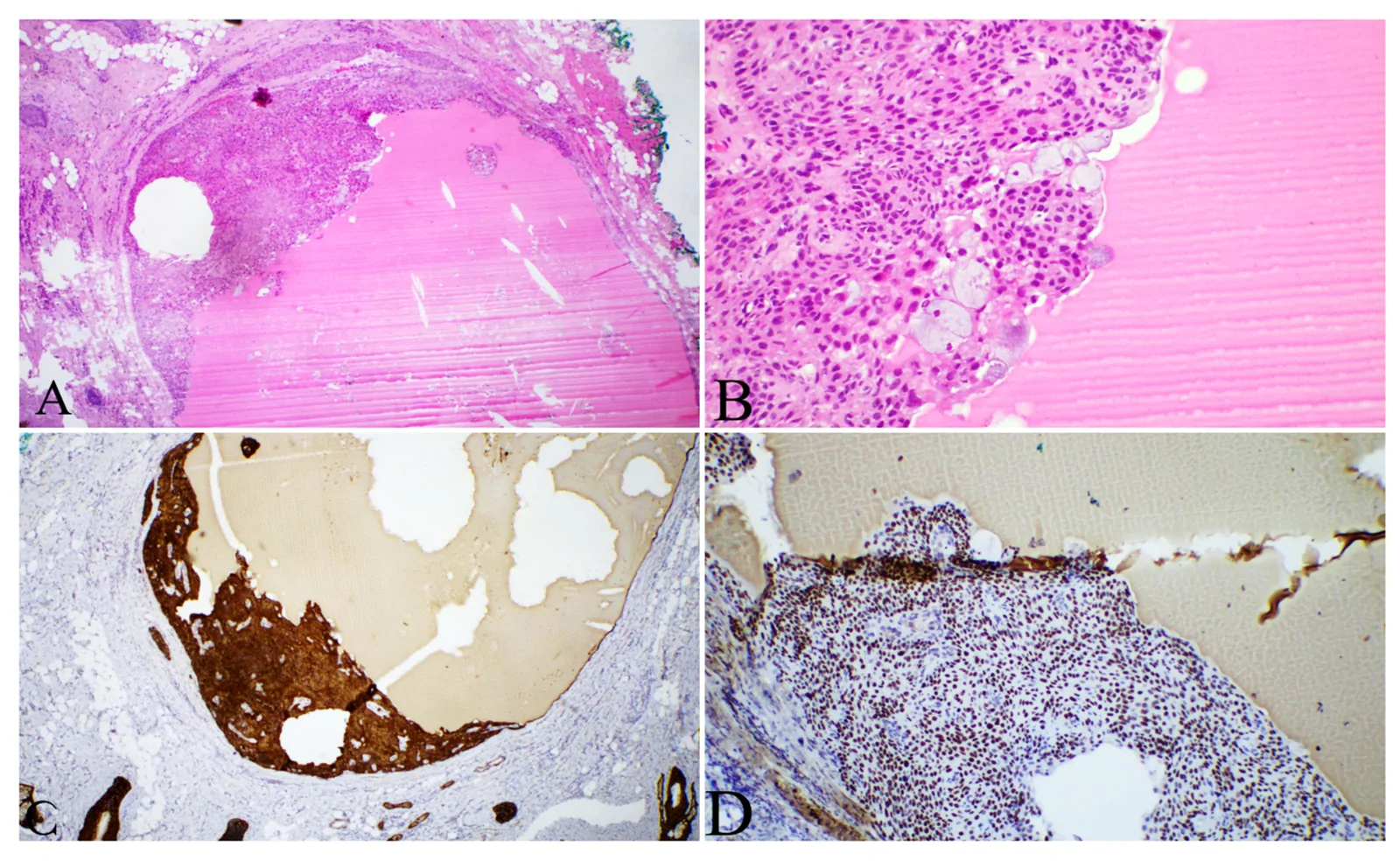
Hidradenoma. (**A**) Solid and cystic tumour proliferation composed of polygonal cells with eosinophilic cytoplasm, 5× objective. (**B**) Polygonal cells with eosinophilic cytoplasm are seen along with foamy cells, with eccentric nuclei and abundant cytoplasm, 20× objective. (**C**) Immunohistochemistry showing positive reaction for marker CK7, 5× objective. (**D**) Immunohistochemistry showing positive reaction for marker p63, retained in the periphery of the tumour, 10× objective.

**Figure 5:**
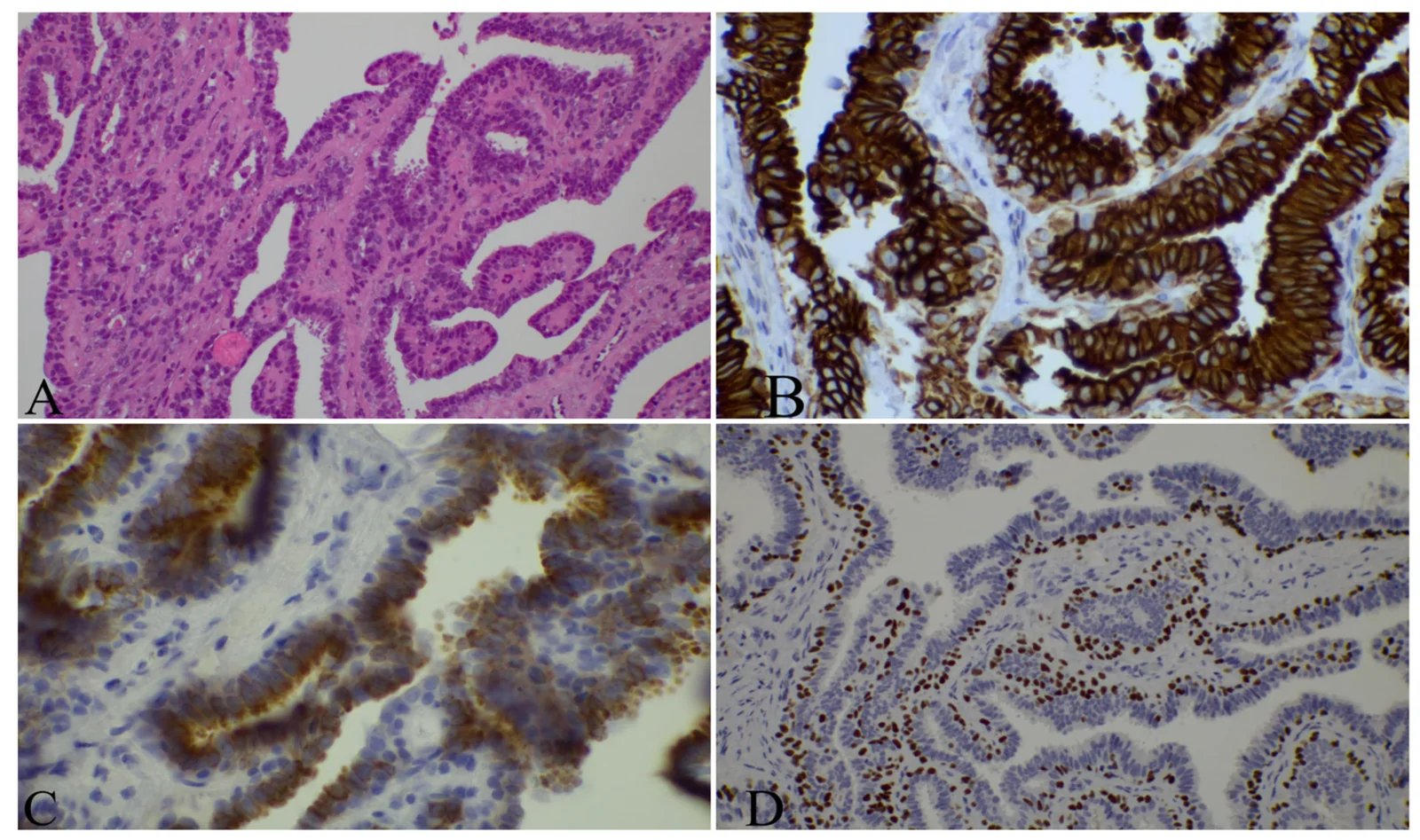
Hidradenoma papilliferum. (**A**) Tumour proliferation with papillary architecture, layered by cells with apocrine features, HE, 20× objective. (**B**) Immunohistochemistry reaction for CK7, 20× objective. (**C**) Immunohistochemistry reaction for EMA, 20× objective. (**D**) Immunohistochemistry reaction for p63, 20× objective (this case was not part of the study cohort; the figure above is included solely for illustrative purposes).

**Figure 6:**
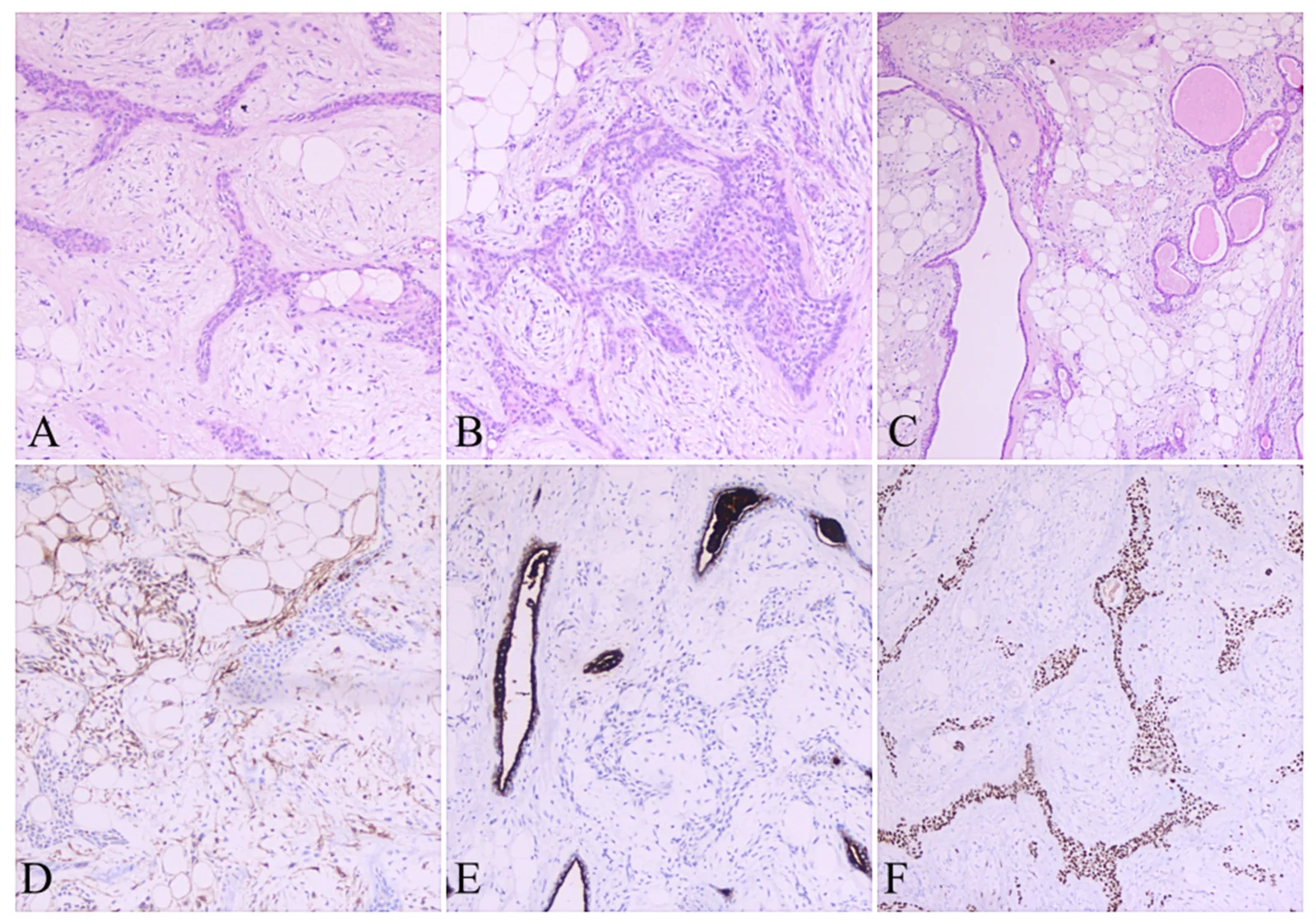
Mixed apocrine tumour. (**A**–**C**) Tumour proliferation is mixed with stromal components, occasionally displaying myxoid or chondroid differentiation, HE, 5× objective. (**D**) Immunohistochemistry reaction showing positive reaction for EMA, 10× objective. (**E**) Immunohistochemistry reaction showing positive reaction for CK7, 10× objective. (**F**) Immunohistochemistry reaction showing positive reaction for p63, 10× objective.

**Figure 7:**
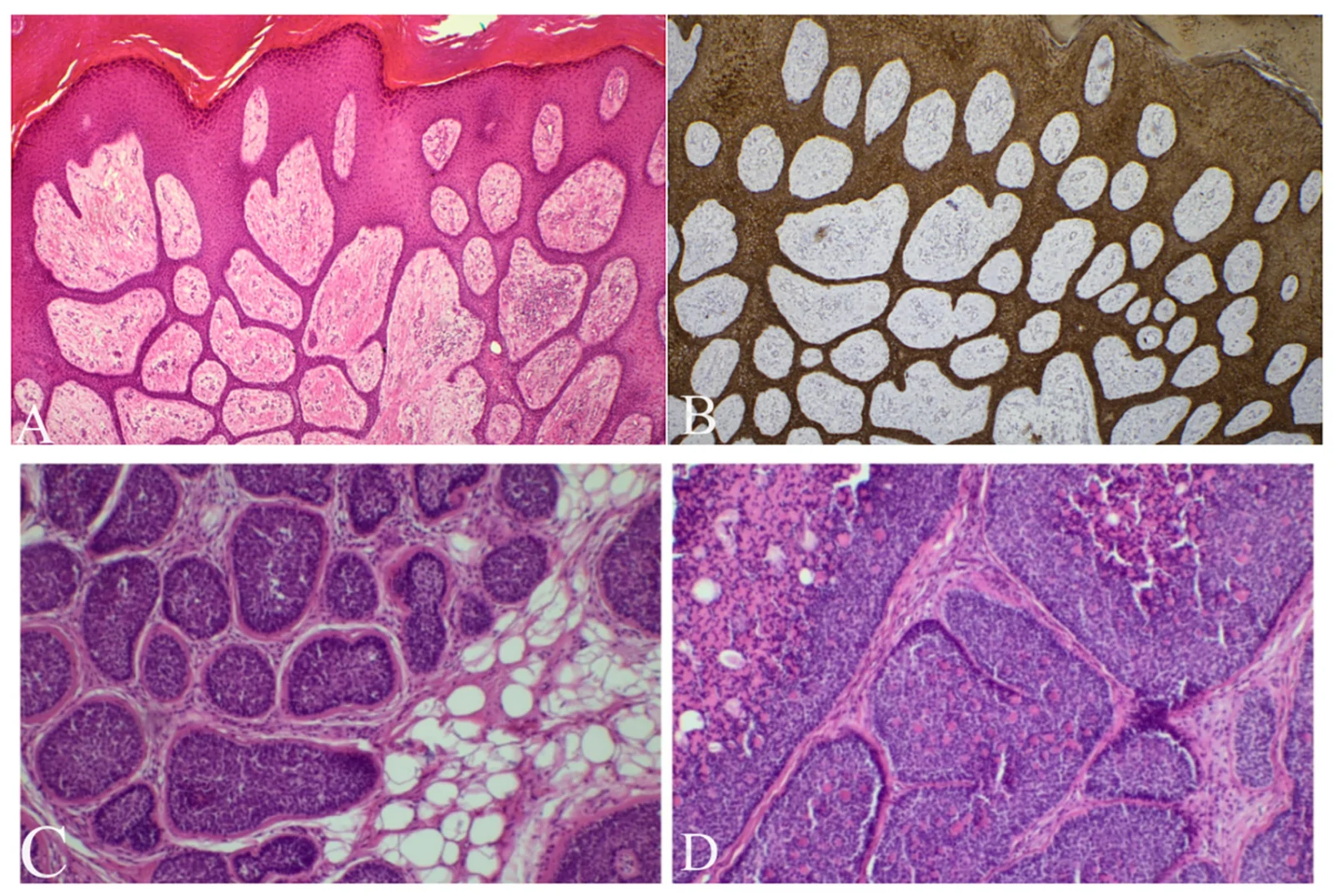
Syringofibroadenoma and Cylindroma. (**A**) Syringofibroadenoma. The tumour is composed of thin epithelial cords that descend into the reticular dermis, creating anastomoses. The tumours are separated by a fibrous stroma with focal inflammation, and within these areas, small and rare ducts can be seen, HE, 10× objective. (**B**) Immunohistochemistry reaction with marker 34BE12 (CTK HMW) shows the branching architecture of the lesions, 10× objective. (**C**,**D**) Cylindroma. (**C**) Tumour nests surrounded by thick, hyalinised fibrous septa, separating them into well-defined lobules, HE, 10× objective. (**D**) The cells present a jigsaw pattern, HE, 10 objective.

**Figure 8:**
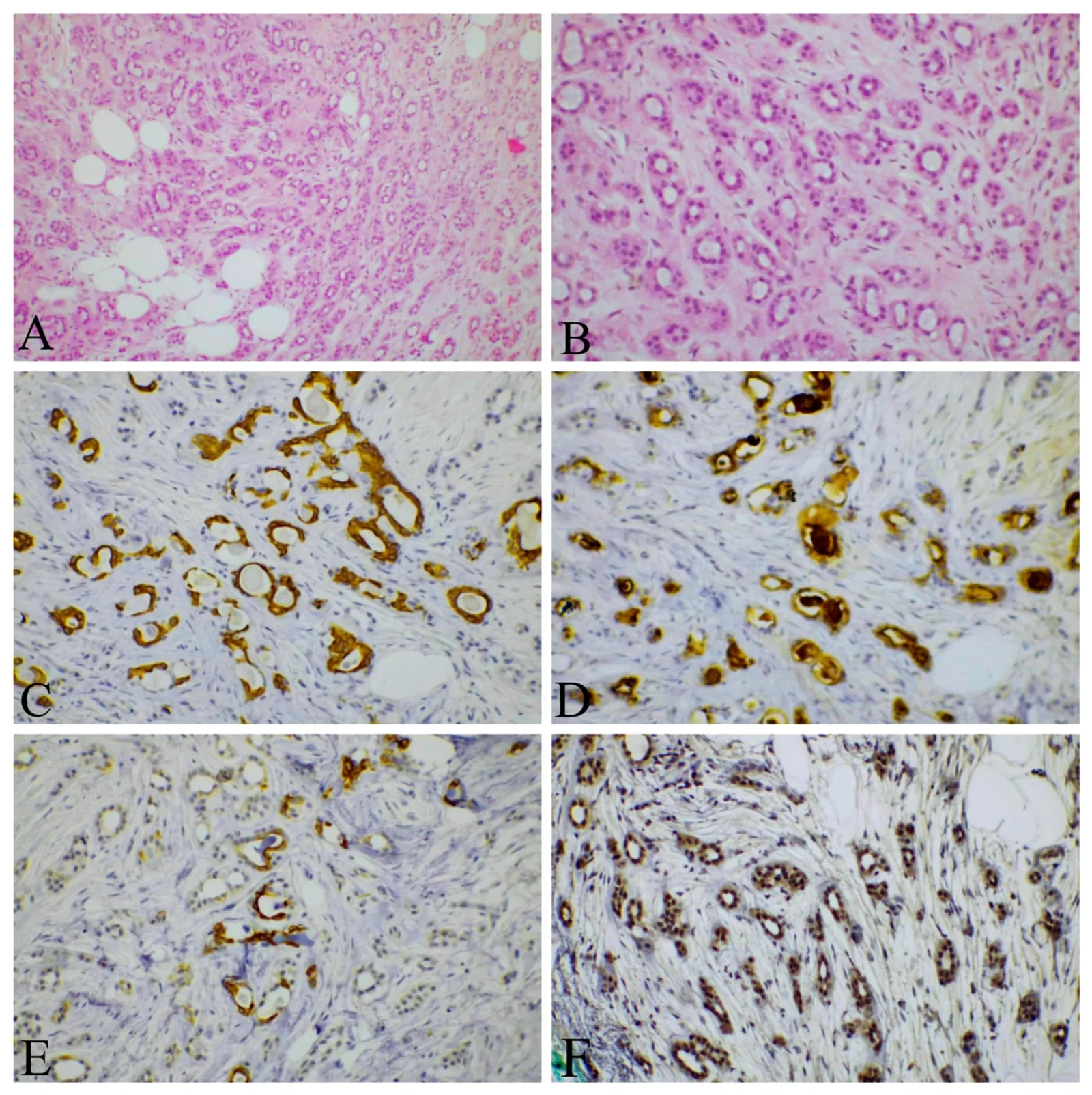
Syringoma. (**A**) Dermal tumour that is composed of multiple tubules of various sizes, some of which are dilated, HE, 10× objective. (**B**) The tubules are layered by a double layer of cells, HE, 20× objective. (**C**–**F**) Immunohistochemistry reaction with marker p63 highlights the basal cells.

Benign sweat gland tumours represented the largest subgroup and included hidrocystomas, spiradenomas, poromas, hidradenomas, mixed apocrine tumour, cylindroma, syringoma, and eccrine syringofibroadenoma. Hidrocystomas were the most frequent lesions and showed classic dermal cystic architecture, while spiradenomas represented the second most common group and showed the typical biphasic cellular pattern. Other benign sweat gland tumours were rare and showed conventional morphological features (Figure 1, Figure 2, Figure 3, Figure 4, Figure 5, Figure 6, Figure 7 and Figure 8).

**Figure 9:**
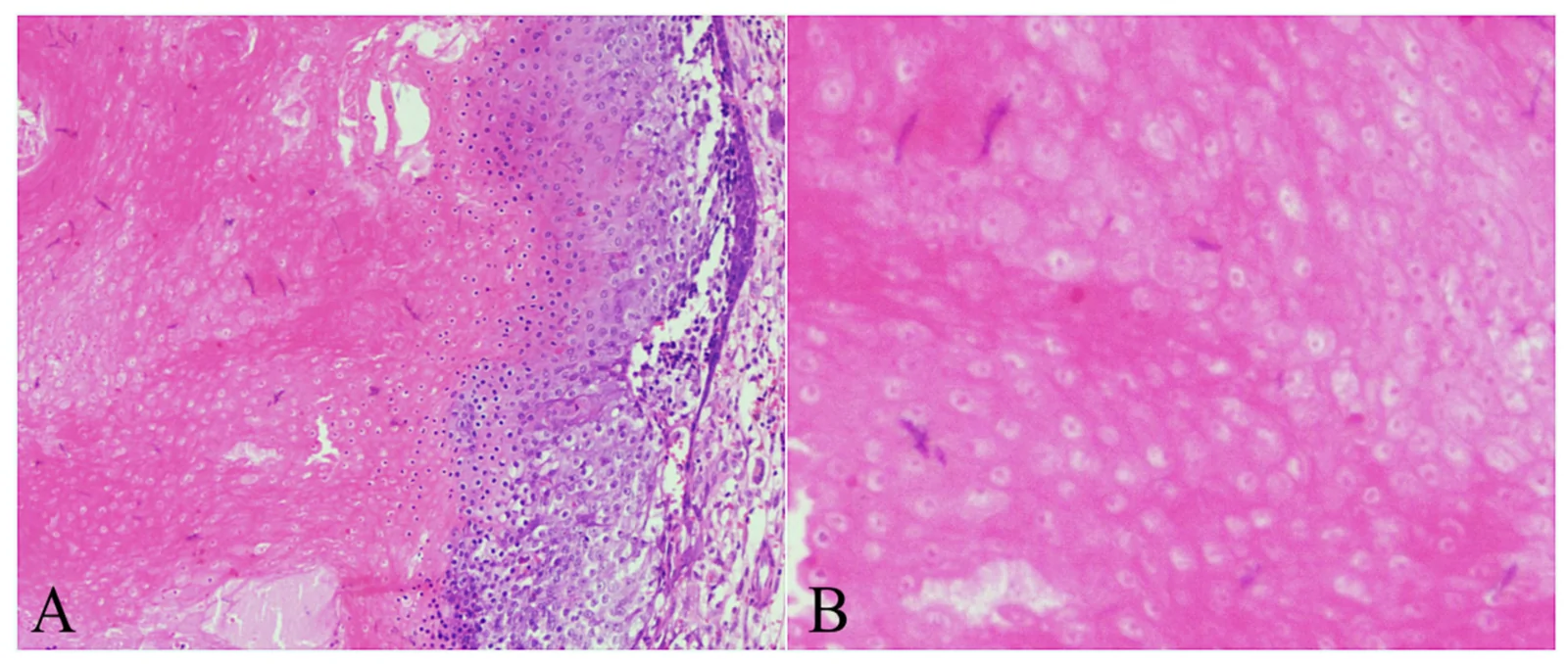
Pilomatrixoma. (**A**) Tumour presents two distinct populations of cells; some are basaloid, round, with pale, reduced cytoplasm, while the others are polygonal, with abundant eosinophilic cytoplasm and absent nuclei (ghost cells). The transition is seen between them as the presence of cells with hyperchromic, pycnotic nuclei; HE, 10×. (**B**) “Ghost cell” population details, HE, 20×.

Benign follicular tumours included trichoblastoma, trichofolliculoma, and pilomatrixoma. These lesions showed classical diagnostic patterns, including follicular germ-like basaloid nests in trichoblastoma, abortive follicular differentiation in trichofolliculoma, and basaloid-to-ghost-cell transition in pilomatrixoma (Figure 9).

Immunohistochemistry was performed when needed for diagnostic confirmation.

#### 3.2.2. Atypical Tumours

Atypical spiradenoma was diagnosed when conventional spiradenoma architecture was retained, but focal cytological atypia, enlarged nuclei, and focally increased mitotic activity were present, with more than 10 mitoses/10 HPF. None of these tumours showed infiltrative growth, extensive necrosis, vascular or lymphatic invasion, or diffuse severe cytological atypia; therefore, the diagnostic threshold for spiradenocarcinoma was not reached. Ki-67 was increased compared with conventional spiradenoma, reaching up to 15% in one recurrent case. The tumour characteristics are shown in Figure 2 and overall features in Table 11.

Seven atypical or malignant tumours were identified in the entire cohort, and their main clinicopathological characteristics are summarised in Table 11. Follow-up information was available for five out of seven patients, although its duration and completeness varied. Among the atypical spiradenomas, one patient remained tumour-free under dermatological surveillance for three years, another had six months of documented tumour-free follow-up with no subsequent information available, while no follow-up data were available for the third patient. Follow-ups for the two porocarcinoma patients were available, one of whom remained under oncological surveillance for two years and the other for more than one year, with no recurrence reported during the documented follow-up. The patient with basal cell carcinoma associated with trichoblastoma remained tumour-free for two years following local radiation therapy. No follow-up data were available for the patient with trichilemmal carcinoma.

Given the small number of cases and the variable duration and completeness of follow-up, these data are presented descriptively and should not be used to draw conclusions regarding prognosis or recurrence risk.

#### 3.2.3. Malignant Tumours

Eccrine porocarcinomas showed certain particularities.

**Figure 10:**
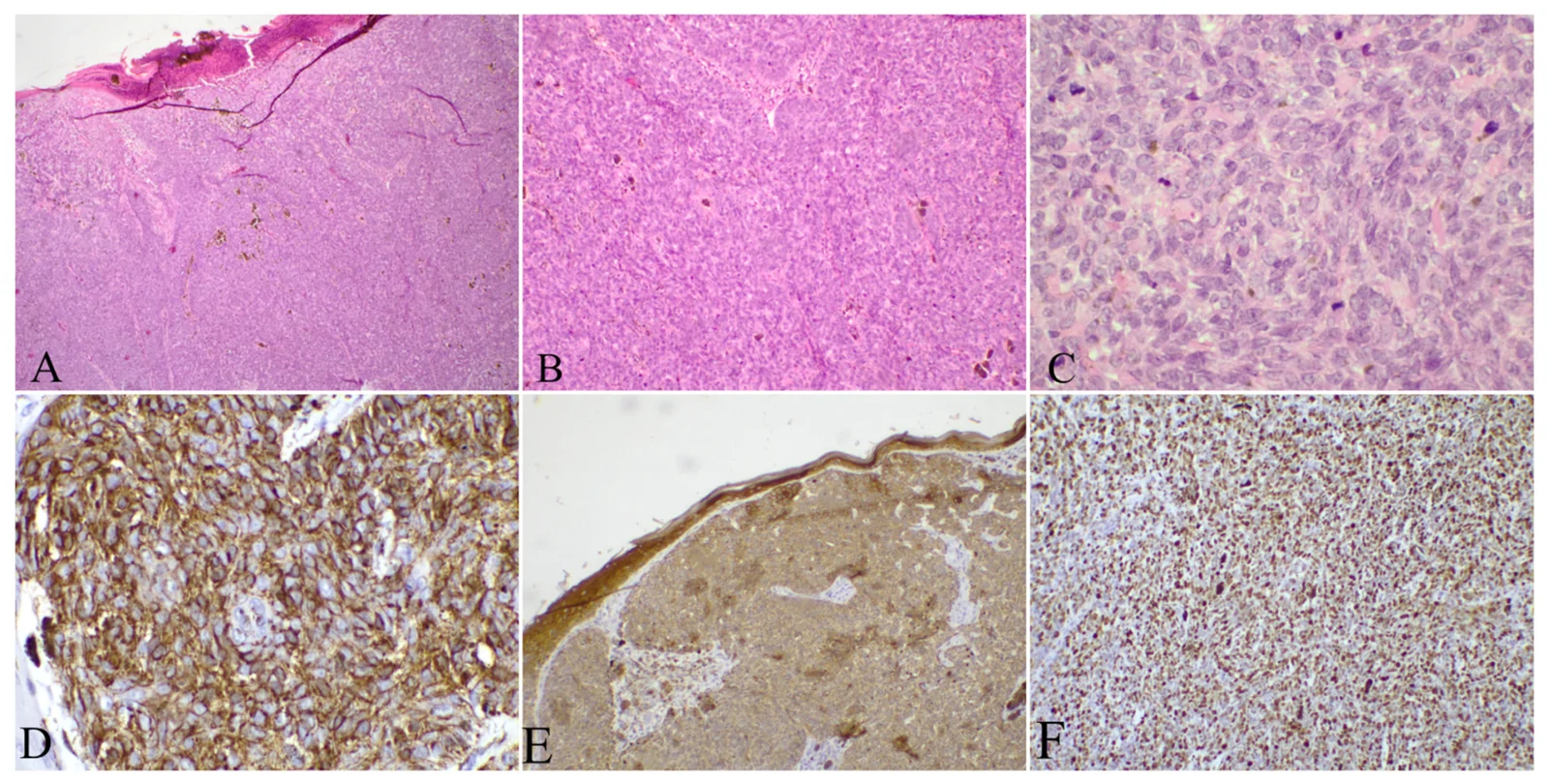
Porocarcinoma. (**A**) Tumour proliferation with solid architecture that ulcerated the epidermis, HE, 5× objective. (**B**) The tumour cells are arranged in plaques, nests, and cords, and present medium-sized, eosinophilic cytoplasm and pleomorphic nuclei, HE, 10× objective. (**C**) Cytological details show nuclear pleomorphism and numerous mitoses, HE, 20× objective. (**D**) Tumour cells show diffuse and intense positive reaction for marker CD117, 20× objective. (**E**) Tumour cells show diffuse positive reaction for CTK AE1/AE3, 5× objective. (**F**) The proliferation index, Ki-67, was expressed in approximately 80% of the tumour cells.

One of the tumours was located on the breast, adjacent to the nipple, which is an uncommon location. The clinical appearance of the lesion did not raise concern for malignancy; instead, it was interpreted as an irritated papilloma. The microscopic examination revealed a solid tumour proliferation with an infiltrative growth pattern. Tumour cells exhibited marked cytological atypia, focal necrosis, and high mitotic activity. The connection with the epidermis, poroid morphology, and immunohistochemistry supported the diagnosis. Immunohistochemistry showed positivity for markers CTK, AE1/AE3, and CD117, and focal positivity for SOX10, while the Ki-67 proliferation index was expressed in 80% of the tumour cells. ER and GATA3 were negative. The tumour was located less than 1 mm from the deep resection margin, and therefore, wide local excision was performed. No residual tumour was identified on the re-excision specimen. The patient did not show signs of recurrent or metastatic disease and is under oncological surveillance. The tumour is shown in Figure 10.

The second porocarcinoma occurred on the scalp of a 40-year-old male patient and was clinically suspected to represent a nevus. Histologically, it showed infiltrative growth, connection with the epidermis, anastomosing cords in the dermis, basaloid and poroid cells with cytological atypia, comedonecrosis, high mitotic activity, CK7 and CD117 positivity, and a Ki-67 proliferation index above 50%. No recurrence has been reported. The patient has remained under oncological surveillance for more than one year.

**Figure 11:**
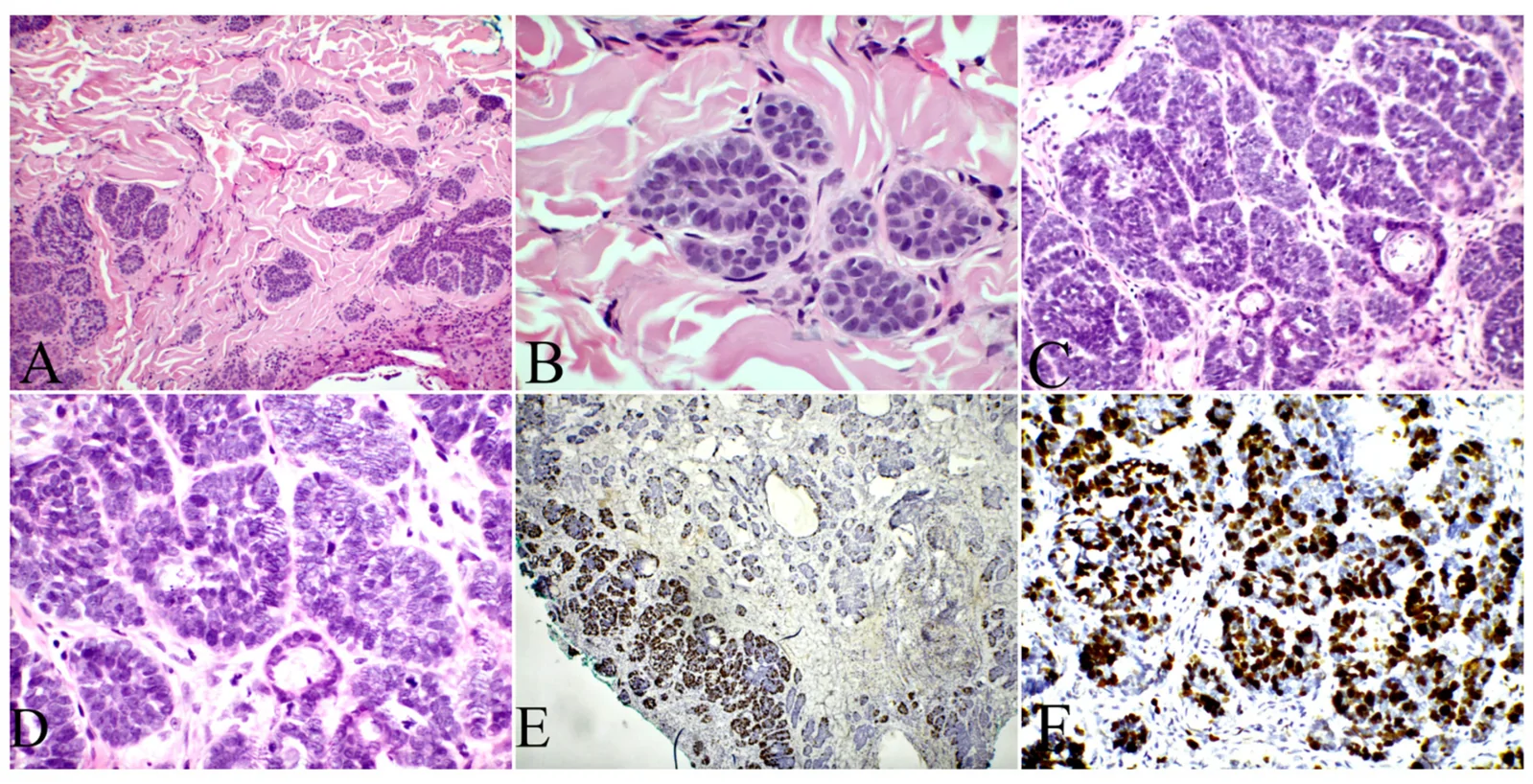
Basal cell carcinoma associated with trichoblastoma. (**A**) Tumour showing classic trichoblastoma features, with nests of basaloid cells distributed in a fibrous stroma, HE, 5× objective. (**B**) Nests of tumour cells corresponding to trichoblastoma. Tumour cells are round, uniform, with no mitoses or atypia visible, HE, 20× objective. (**C**) At the periphery of the tumour, a solid distribution of tumour cells is seen, showing clear palisading features with enlarged, pleomorphic nuclei. Numerous mitoses are seen, HE, 10× objective. (**D**) Tumour cells corresponding to the basal cell carcinoma component, showing atypia and mitoses, HE, 20× objective. (**E**) Proliferation index Ki-67 showing different expression in the benign versus the carcinoma component, 5× objective. (**F**) Proliferation index Ki-67 expressed predominantly in basal cell carcinoma, 20× objective.

Basal cell carcinoma associated with trichoblastoma is a rare event; in our cohort, it affected an elderly male patient and was located on the eyelid. The patient experienced three recurrences until, eventually, tumour-free margins were obtained. The microscopic examination showed a tumour with conventional trichoblastoma features. One of the examined fragments showed nodular basal cell carcinoma. The carcinoma component presented nodular enlargement with more irregular architecture, peripheral palisading, and high mitotic activity. The Ki-67 index was increased in the carcinoma component and low in the trichoblastoma component. The patient is currently tumour-free. The characteristics of this tumour are shown in Figure 11.

This case was interpreted as basal cell carcinoma, most likely arising in association with trichoblastoma. The diagnosis was supported by the coexistence of a conventional trichoblastoma component and a morphologically distinct carcinoma component showing architectural irregularity, peripheral palisading, increased mitotic activity, and a higher Ki-67 proliferation index. Although a collision tumour cannot be entirely excluded, the intimate association between the two components supports the interpretation of carcinoma arising in association with trichoblastoma.

Trichilemmal carcinoma occurred in a 66-year-old male patient, with no relevant clinical history and was clinically suspected to present a basal cell carcinoma on the thorax. Histological examination revealed a predominantly solid tumour proliferation composed of well-demarcated lobules surrounded by connective tissue stroma. The tumour cells were medium to large, polygonal to oval, with abundant eosinophilic cytoplasm and enlarged vesicular nuclei with prominent centrally located nucleoli. Cytological atypia and mitotic activity were identified, together with areas of surface keratinisation. A polymorphous inflammatory infiltrate was present at the tumour-stromal interface. Immunohistochemical evaluation showed a Ki-67 proliferation index of approximately 40%. The morphological and immunohistochemical findings supported the diagnosis of infiltrative trichilemmal carcinoma. The tumour was completely excised, with tumour-free resection margins. No additional information regarding subsequent treatment or postoperative follow-up was available.

## 4. Discussion

### 4.1. Epidemiological and Pathological Characteristics of Primary Adnexal Tumours

The main contribution of this study is the documentation of a regional Eastern European cohort of cutaneous adnexal tumours diagnosed in routine pathology practice. While adnexal tumours are well recognised histopathologically, cohort-level data from this geographic area remain limited.

The demographic analysis presented in Table 1 showed that out of the total of 82 primary adnexal tumours included in the study, patients ranged in age from 24 to 90 years. Females accounted for 62.19% of cases (Table 1), whereas males represented 37.81%, without a significant sex difference (*p* = 0.1145) nor in age difference (*p* = 0.2747; Table 1), even though female patients presented a lower mean age, 56 ± 18 years, compared to males, 60 ± 14 years. The female predominance observed in our cohort is consistent with several previous reports, in which men accounted for less than 50% of the studied population [1,2], indicating a higher prevalence of adnexal tumours among women. In terms of age analysis, in a series of 65 patients with adnexal tumours, reported ages ranged from 8 to 88 years, with a mean age of 46.15 ± 21.8 years [2] notably younger than our series, whereas in a larger series of 1016 adnexal tumours, lesions were more frequently in patients aged between 51 and 70 years, closer to the mean ages seen in our cohort [1].

According to the year of diagnosis (Table 1), the highest number of cases was observed in 2024 (30.49%), followed by 2022 (17.07%) and 2023 (15.85%). The annual case distribution did not differ significantly (*p* = 0.0759), with mean age remaining comparable across the study period (*p* = 0.6490), with a slight tendency towards a decrease. This observation might be attributed to the indirect effect of the COVID-19 pandemic, where the health system was on hold and procedures were postponed, resulting in delays in case management. On the 8th of March 2022, the Romanian health system returned to full capacity, and therefore the constant increase in cases from 2022 to 2024 might be a result of this effect also seen internationally [3,4,5].

In terms of topographic distribution (Table 2), most lesions were observed in the head and neck region (70.73%), followed by the upper limb (12.19%), thorax (8.54%), lower limb (7.32%), and one unspecified location. The distribution of anatomical sites according to sex was not significantly different (*p* = 0.8887). The subanalysis of the head and neck region case distribution, even though insignificant (*p* = 0.1614; Table 2), showed that the ocular region represented the most frequently affected subsite with 60.34% of cases. The next subsite was the scalp with 22.41%, followed by the facial region with 6.90%, the cervical region with 5.17%, the nasal region with 3.45%, and the least affected area being the labial region with only 1.72% of cases. The strong predilection seen in the head and neck area is also observed in other studies [6]. This concordance likely reflects the higher density of pilosebaceous and eccrine/apocrine structures found in this region, from which most adnexal tumours arise. The predominance of the ocular subsite within the head and neck subgroup might be attributed to the density of adnexal glands around the eyelid and periorbital skin [7,8].

The histopathological spectrum of primary adnexal tumours presented in Table 3 and Table 4 showed that most lesions (79.27%; *n* = 65) were of sweat gland origin, whereas hair follicle tumours accounted for a smaller proportion (20.73%; *n* = 17). Among sweat gland tumours, eccrine hidrocystoma was the most frequent lesion observed (23.07%; *n* = 15), while cylindroma (1.54%; *n* = 1) and eccrine syringofibroadenoma (1.54%; *n* = 1) were less frequent. Among hair follicle tumours (Table 3), trichoblastoma (41.18%; *n* = 7) and pilomatrixoma (35.29%; *n* = 6) were the most common, trichofolliculoma was seen in only two patients (11.76%; *n* = 2), while both trichoblastoma associated with basal cell carcinoma (5.88%; *n* = 1) and trichilemmal carcinoma (5.88%; *n* = 1) were the least observed. The predominance of sweat gland tumours in our series contrasts with several other reported cohorts, in which sebaceous or follicular tumours often dominate. In the South India study by Bhat et al. [6], sweat gland tumours formed the largest group as well, involving 55% of cases with only 10% of each apocrine and sebaceous gland tumours, a pattern relatively concordant with our findings. This relative excess of sweat gland-derived lesions, especially eccrine hidrocystoma found in our study, is likely linked to the ocular predominance in the topographic analysis, given the density of eccrine and apocrine structures in that region.

Biological behaviour (Table 4) did not differ significantly between male and female patients (*p* = 0.2650). However, benign tumours were more frequent among females (94.12%) than males (87.10%), whereas malignant tumours accounted for 1.96% in females and 9.68% in males, respectively. The predominance of benign lesions is widely known in the literature, with several retrospective studies reporting that 80.36% to 95.40% of CATs in their cohorts were benign [2,9,10,11].

Tumour lineage showed no significant association with sex (*p* = 0.5466), indicating a rather heterogeneous distribution of these lesions between male and female patients. Likewise, no significant association was observed between tumour lineage and biological behaviour of tumours (*p* = 0.2787; Table 5), with malignant tumours equally distributed between hair follicle and sweat gland differentiation in the present cohort. Previous studies have reported heterogeneous findings regarding the predominant lineage of malignant CATs. Compared to our results, both a European study and the study by Melekber Ç. Özkan et al. found that malignant adnexal tumours more commonly exhibit sweat gland differentiation [12,13].

Patient age differed significantly according to biological behaviour (*p* = 0.0058; Table 6). Patients with atypical tumours had the lowest mean age (28 ± 4 years), followed by those with malignant tumours (52 ± 14 years), whereas benign tumours mainly occurred in patients with a mean age of 59 ± 16 years. The younger age observed in the atypical group should be interpreted cautiously due to the limited number of cases. This finding may reflect the specific histological composition of the cohort, with most benign adnexal tumours having a rather indolent, slow-growing period before excision [14]. Malignant tumours, however, predominantly affect middle-aged and older adults, with other studies reporting a median age of onset at ≥60 years [13]. Overall, the age distribution observed in the present study is consistent with the heterogeneous epidemiological patterns reported for CATs [13,15]. Further analysis showed that mean patient age did not differ significantly between hair follicle and sweat gland tumours (*p* = 0.1639). Likewise, median age did not differ according to general anatomical topography (*p* = 0.3932) even though patients with lower limb lesions presented a lower median age (median: 55 years; IQR [31–59 years]) compared to the highest median age that was observed among patients presenting upper limb lesions (median: 67 years; IQR [34–82 years]). However, within the head and neck region (Table 6), patient age varied significantly among anatomical subsites (*p* = 0.0361), with the lowest mean age being found at a cervical level, 38 ± 22 years, compared to the rest of the sites where the mean age varied between 53 ± 19 years in the scalp and 61 ± 11 years in those with ocular lesions.

Neither tumoural volume nor specimen volume differed significantly between male and female patients (*p* = 0.1317 and *p* = 0.2068, respectively), although males exhibited higher median values for both excision specimen volume (median: 254.3 mm^3^; IQR [29.05–1099 mm^3^]), and tumour volume (median: 125.6 mm^3^; IQR [10.99–374.2 mm^3^]). Previous studies have described associations between tumour diameters and malignant lesions, rather than gender-related differences in terms of size [16], but no volumetric analysis has been reported. Pujani M. et al. analysed 25 adnexal tumours and over 50% of them had a maximum diameter under 1.5 cm and did not observe any sex-related significant differences [17].

Specimen volume differed significantly according to general topography (*p* = 0.0003), with the largest median volume observed in lesions of the upper limb (median: 1444.0 mm^3^; IQR [268.6–3526.0 mm^3^]), whereas the head and neck lesions exhibited the smallest median specimen volume (median: 78.5 mm^3^; IQR [18.84–314.0 mm^3^]). In contrast, tumour volume did not differ significantly according to general topography (*p* = 0.5538), even though thoracic (median: 30.09 mm^3^) and head and neck (median: 39.25 mm^3^) areas exhibited smaller tumour volumes. These results extend previous research, which has reported a predominance of adnexal tumours in the head and neck region but did not evaluate the volumetric differences according to anatomical distribution of the lesions [16,18]. A reasonable explanation for smaller specimen volumes would be the increased incidence of lesions in cosmetically sensitive areas, facilitating earlier recognition and clinical assessment. In addition, surgical management of head and neck lesions is frequently influenced by functional and aesthetic considerations, favouring tissue-sparing excisions whenever oncologically appropriate [19,20].

Further analysis of the head and neck lesions demonstrated significant differences in specimen volume among anatomical subsites (*p* < 0.0001). Cervical lesions exhibited the largest median volume (median: 1047 mm^3^; IQR [241.8–2638 mm^3^]), whereas ocular lesions had the smallest median specimen volume (median: 19.89 mm^3^; IQR [6.67–60.45 mm^3^]). Although tumour volume did not differ significantly among head and neck subsites (*p* = 0.0684), it followed a distribution similar to excision specimens. To our knowledge, previous studies have primarily described the anatomical distribution of CATs, whereas volumetric differences between head and neck subsites have not been investigated [13,14,18]. Studies regarding other skin tumours showed limited excision margins for the ocular area because of the functional and cosmetic sensitivity of the region, and Mohs is recommended to preserve healthy tissue [19,20,21].

Further volumetric analysis showed no significant differences regarding tumours according to biological behaviour (*p* = 0.2546) nor between sweat gland and hair follicle differentiation (*p* = 0.5880). Previous studies have primarily compared the frequency and histological spectrum of follicular and sweat gland tumours, whereas differences in tumour dimensions according to adnexal differentiation have rarely been investigated [13,18]. Additionally, no significant correlations were observed between patient age and specimen volume (*p* = 0.1319) or tumour volume (*p* = 0.3280), indicating that lesion growth is unlikely to be determined by chronological ageing. Likewise, neither tumour (*p* = 0.7295) nor specimen volume (*p* = 0.1445, Table 8) correlated significantly with the diagnosis year, providing no evidence of a temporal change in volumetric measurements during the study period, despite changes in diagnostic activity over time. The absence of a temporal trend may also indicate that tumour dimensions remained relatively stable throughout the study period, despite changes in diagnostic activity over time.

No significant differences in surgical margin status were observed between male and female patients (*p* = 0.6992), although among cases with tumour-free margins, 41 (80.39%) occurred in female patients. This finding is consistent with previous clinical and histopathological studies of CATs, which have not identified patient sex as a determinant of surgical outcomes or margin status, with surgical management being guided mostly by tumour characteristics rather than patient demographics [13,16]. Topographical localisation, however, significantly influenced surgical margin status (*p* = 0.0069), with the highest proportion of free margins observed in head and neck lesions, whereas two of the seven thoracic lesions (28.57%) had margins < 1 mm. Similar observations have been reported for head and neck skin tumours, where careful preoperative planning and anatomically adapted excisions are employed to maximise complete tumour removal while preserving function and cosmesis [22]. Although no significant association was identified between surgical margin status and head and neck sublocalisation (*p* = 0.0720), periocular lesions exhibited the highest proportion of free margins (97.14%; *n* = 34) among the head and neck subgroup. This finding is consistent with current recommendations favouring meticulous margin-controlled surgery, including Mohs micrographic surgery when appropriate, in cosmetically and functionally sensitive regions, allowing complete tumour excisions despite limited tissue availability [22,23]. Tumour lineage was also significantly associated with surgical margin status (*p* = 0.0031). Hair follicle tumours accounted for all infiltrated-margin cases observed in the present cohort, whereas sweat gland tumours were predominantly associated with tumour-free resections. Given the small number of infiltrated lesions, this finding should be interpreted cautiously and requires confirmation in larger series.

Biological behaviour was significantly associated with surgical margin status (*p* = 0.0003), with benign tumours showing the highest proportion of complete excisions (88.00%; *n* = 66), whereas malignant lesions presented more frequently with close or infiltrated margins, and atypical lesions’ margins could not be evaluated. These findings are consistent with biological characteristics of malignant CATs, which typically exhibit a more infiltrative growth pattern and less well-defined tumour borders than their benign counterparts, making complete surgical excision more challenging. Accordingly, complete excision with histologically negative margins remains the cornerstone of treatment for malignant adnexal neoplasms, and margin-controlled techniques such as Mohs micrographic surgery are recommended for selected high-risk lesions to reduce the likelihood of residual disease and local recurrence [24]. In contrast, margin status was not significantly associated with tumour volume (*p* = 0.5559), specimen volume (*p* = 0.0901), or patient age (*p* = 0.2294). Like previous research, these findings suggest that successful surgical clearance may be influenced by tumour biological behaviour and growth pattern rather than lesion size or patient-related characteristics [13].

### 4.2. Histopathology of Primary Adnexal Tumours

Our findings confirm the predominance of benign lesions, which accounted for more than 90% of cases, but also underline several diagnostically relevant rare lesions, including atypical spiradenomas, two eccrine porocarcinomas, a trichilemmal carcinoma, and a basal cell carcinoma most likely arising in association with trichoblastoma. This distribution supports previous clinicopathological observations that malignant adnexal tumours remain rare despite the broad morphological diversity of this group [25,26].

In our cohort, sweat gland tumours were the most common, with hidrocystomas the most frequent subtype. Spiradenomas represented the second most common subtype, illustrating the broad morphological spectrum encountered in routine dermatopathology practice. Their heterogeneous anatomical distribution and characteristic biphasic cellular populations confirm that architectural pattern recognition remains essential for diagnosis despite overlapping cytological features [27,28].

The two eccrine porocarcinomas represented important diagnostic pitfalls. The breast/nipple-adjacent lesion was particularly challenging because this location raises the differential diagnoses of primary breast carcinoma, mammary Paget disease, metastatic carcinoma, and other adnexal carcinomas. In this case, the absence of ER and GATA3 expression argued against conventional breast carcinoma, while the epidermal connection, poroid morphology, ductal differentiation, cytokeratin expression, CD117 positivity, marked cytological atypia, infiltrative growth, necrosis, and high Ki-67 index supported the diagnosis of eccrine porocarcinoma. The second porocarcinoma, located on the scalp and clinically suspected to be a nevus, further illustrates the nonspecific clinical appearance of adnexal malignancies and the central role of histopathological examination [29].

Adnexal tumours continue to represent a histopathological diagnostic challenge due to shared architectural patterns among entities such as spiradenoma, hidradenoma, cylindroma, and poroma. In this context, immunohistochemistry served mainly as an adjunctive tool, supporting lineage differentiation rather than establishing a definitive diagnosis. Markers such as CK7, EMA, and p63 proved useful in confirming ductal or basal cell differentiation, yet the immunophenotypic overlap between tumours highlights the limitations of relying solely on ancillary techniques. Correlation with the patient’s clinical history and establishing the primary or secondary origin of the tumours are also essential, since adnexal carcinomas can frequently metastasise to the skin or mimic other neoplasms. Our findings highlight the widely accepted view that meticulous morphological evaluation remains the cornerstone of adnexal tumour classification. Emerging markers may support the diagnosis of selected adnexal tumours, but their role remains complementary and should be interpreted according to tumour type and morphology [16,28,30,31].

The identification of atypical spiradenomas in our cohort provides further evidence of a biological continuum between benign adnexal tumours and their malignant counterparts. Increased proliferative activity, reflected by higher Ki-67 expression, together with focal cytological atypia, represented key features suggesting progression toward malignancy. Similarly, eccrine porocarcinoma displayed an infiltrative growth pattern, significant cytological atypia, and highly elevated Ki-67 values. These illustrate the importance of integrating histological and immunohistochemical findings when assessing tumours with ambiguous morphology [32].

Follicular tumours identified in our cohort included trichoblastoma, trichofolliculoma, and pilomatrixoma, which showed classical histological patterns and low proliferative indices, consistent with their typically indolent behaviour. However, the presence of basal cell carcinoma in association with trichoblastoma underscores the need for extensive sampling in lesions showing nodular enlargement or increased mitotic activity. This observation highlights that carcinoma may occur in association with otherwise benign follicular adnexal tumours, although such events are rare [33].

The morphological heterogeneity observed across our cohort supports the concept that adnexal tumours represent a diagnostic challenge. The coexistence of cystic, solid, and mixed architectural patterns within the same lineage may complicate classification, even in excisional specimens, particularly when tumours are small, heterogeneous, recurrent, or only partially represented in examined sections. In this context, clinicopathological correlation and awareness of anatomical predilections become essential tools for accurate diagnosis [29,34].

#### Study Limitations

This study has several limitations that should be acknowledged. The retrospective design and single-centre nature of the cohort may introduce selection bias, particularly in a tertiary referral setting where complex or unusual lesions are more likely to be evaluated. Furthermore, molecular analyses were not performed, which limits the ability to correlate with emerging genetic data increasingly contributing to adnexal tumour classification.

Despite these limitations, the relatively large number of cases and detailed histopathological evaluation provide valuable insight into the morphological spectrum of adnexal neoplasms encountered in routine practice. The small number of atypical and malignant tumours limit the statistical power of subgroup analyses. Consequently, analyses involving biological behaviour should be considered exploratory, and both significant and non-significant findings derived from small subgroups should be interpreted cautiously.

The volumetric analysis also presents some limitations. Tumour and specimen volumes were estimated from three dimensions given during gross examination and the ellipsoid approximation. Furthermore, complete dimensional data were not available for all cases, particularly for tumour volume. Cases with incomplete measurements were excluded, resulting in smaller samples for volumetric analyses and potentially introducing selection biases.

Follow-up information for some atypical and malignant lesions was limited and heterogeneous, precluding reliable assessment of recurrence rates, disease-free survival, or long-term prognosis. Immunohistochemistry was performed according to morphological and differential diagnostic considerations rather than systematically across the entire cohort.

Our findings emphasise that while immunohistochemistry and proliferative markers provide valuable supporting information, they cannot replace careful morphological assessment. Accurate diagnosis requires integration of architectural patterns, cytological features, anatomical localisation, and immunophenotype. The broad spectrum observed in this series highlights the continued relevance of histopathological criteria in the era of expanding ancillary techniques.

## 5. Conclusions

Our findings contribute to the limited data available from Eastern European populations and highlight the importance of integrating morphological features with epidemiological, clinical, and topographical particularities.

Our study highlights the head and neck region was the predominant site for primary cutaneous adnexal tumours, particularly the periocular area, while sweat gland-derived lesions, especially eccrine hidrocystoma, represented the most common histological subtype.

Most tumours followed a benign course, although numerically higher proportion of malignant tumours was observed among male patients. Atypical lesions were observed in younger individuals in our cohort; however, given the very small number of atypical cases (*n* = 3), this descriptive observation should be interpreted cautiously.

Specimen volumes varied by anatomical site, with head and neck lesions requiring smaller specimens, likely reflecting the cosmetic and functional surgical constraints of the region. Malignant and atypical tumours were associated with less favourable margin status, underscoring the need for careful surgical planning in these cases.

The higher number of cases observed in 2024 may reflect the resolution of care delays following pandemic-related disruptions to the healthcare system rather than a true rise in disease incidence.

These findings emphasise that anatomical location, histological subtype, and biological behaviour should be jointly considered when planning surgical management and follow-up of adnexal tumours, particularly those arising in the periocular and head and neck regions.

## Figures and Tables

**Table 1:** Case and age distribution according to sex and year of diagnosis.

Variables		*n*	%	*p*	Mean (Years)	SD (Years)	*p*
Sex	Female	51	62.19%	0.1145	56	±18	0.2747
	Male	31	37.81%		60	±14	
Year of diagnosis	2018	5	6.10%	0.0759	66	±16	0.6490
	2019	6	7.32%		64	±22	
	2020	5	6.10%		54	±13	
	2021	9	10.98%		58	±16	
	2022	14	17.07%		53	±16	
	2023	13	15.85%		59	±10	
	2024	25	30.49%		55	±21	
	2025	5	6.10%		62	±5	

**Table 2:** Adnexal tumours topographic distribution.

Variables		*n* (Total)	% (Total)	Female		Male		*p*
				*n*	%	*n*	%	
General topography	Head and neck	58	70.73%	35	60.34	23	39.66	0.8887
	Upper limb	10	12.19%	7	70.00	3	30.00	
	Lower limb	6	7.32%	3	50.00	3	50.00	
	Thorax	7	8.54%	5	71.43	2	28.57	
	Unspecified	1	1.22%	1	100.00	0	0.00	
Head and neck region	Scalp	13	22.41%	6	46.15	7	53.85	0.1614
	Facial	4	6.90%	1	25.00	3	75.00	
	Ocular	35	60.34%	25	71.43	10	28.57	
	Nasal	2	3.45%	1	50.00	1	50.00	
	Cervical	3	5.17%	2	66.67	1	33.33	
	Labial	1	1.72%	0	0.00	1	100.00	

**Table 3:** Adnexal tumours distribution according to sex, biological behaviour and lineage.

Diagnosis	Lineage	Biological Behaviour	Female		Male	
			*n*	%	*n*	%
Eccrine hidrocystoma	Sweat gland	Benign	11	73.33	4	26.67
Apocrine hidrocystoma	Sweat gland	Benign	4	57.14	3	42.86
Hidrocystoma NOS	Sweat gland	Benign	9	69.23	4	30.77
Mixed eccrine-apocrine hidrocystoma	Sweat gland	Benign	2	100.00	0	0.00
Eccrine spiradenoma	Sweat gland	Benign	6	66.67	3	33.33
Atypical spiradenoma	Sweat gland	Atypical	2	66.67	1	33.33
Eccrine poroma	Sweat gland	Benign	2	40.00	3	60.00
Eccrine porocarcinoma	Sweat gland	Malignant	1	50.00	1	50.00
Hidradenoma	Sweat gland	Benign	1	33.33	2	66.67
Mixed apocrine tumour	Sweat gland	Benign	1	50.00	1	50.00
Cylindroma	Sweat gland	Benign	1	100.00	0	0.00
Eccrine syringofibroadenoma	Sweat gland	Benign	1	100.00	0	0.00
Syringoma	Sweat gland	Benign	1	50.00	1	50.00
Trichoblastoma	Hair follicle	Benign	4	57.14	3	42.86
Trichofolliculoma	Hair follicle	Benign	0	0.00	2	100.00
Pilomatrixoma	Hair follicle	Benign	5	83.33	1	16.67
Basal cell carcinoma associated with trichoblastoma	Hair follicle	Malignant	0	0.00	1	100.00
Trichilemmal carcinoma	Hair follicle	Malignant	0	0.00	1	100.00
Total	-	-	51	62.19	31	37.81

**Table 4:** Distribution of tumour biological behaviour and lineage according to sex.

Variables		Total		Female		Male		*p*
		*n*	%	*n*	%	*n*	%	
Biological behaviour	Benign	75	91.46	48	94.12	27	87.10	0.2650
	Malignant	4	4.88	1	1.96	3	9.68	
	Atypical	3	3.66	2	3.92	1	3.23	
Lineage	Hair follicle	17	20.73	9	17.65	8	25.81	0.5466
	Sweat gland	65	79.27	42	82.35	23	74.19	

**Table 5:** Association between tumour biological behaviour and lineage.

	Category	Hair Follicle (*n*)	Sweat Glands (*n*)	*p*
Biological behaviour	Benign	15	60	0.2787
	Malignant	2	2	
	Atypical	0	3	

**Table 6:** Patient age according to biological behaviour, tumour lineage, and topography.

Age	**Variables**		***n***	**Mean (Years)**	**SD (Years)**		***p***
	Biological behaviour	Benign	75	59	±16		0.0058
		Malignant	4	52	±14		
		Atypical	3	28	±4		
	Origin	Hair follicle	17	51	±22		0.1639
		Sweat gland	65	59	±15		
	**Variables**		***n***	**Median (years)**	**Q1 (years)**	**Q3 (years)**	***p***
	General Topography	Head and neck	58	62	49	69	0.3932
		Upper limb	10	67	34	82	
		Lower limb	6	55	31	59	
		Thorax	7	62	40	69	
	**Variables**		***n***	**Mean (years)**	**SD (years)**		***p***
	Head and Neck Topography	Scalp	13	53	±19		0.0361 *
		Facial	4	60	±13		
		Ocular	35	61	±11		
		Nasal	2	60	±8		
		Cervical	3	38	±22		
		Labial	1	72	±0		

* Labial and nasal lesions were excluded from the analysis due to low frequency.

**Table 7:** Tumour and surgical specimen volumes according to sex, topography, and histopathological characteristics.

Variables		*n*	Median (mm^3^)	Q1 (mm^3^)	Q3 (mm^3^)	*p*
Specimen volume	Female	48	113.6	19.36	490.6	0.2068
	Male	30	254.3	29.05	1099	
Tumour volume	Female	25	40.30	6.28	162.2	0.1317
	Male	19	125.6	10.99	374.2	
Specimen volume	Head and neck	55	78.5	18.84	314.0	0.0003
	Upper limb	10	1444.0	268.6	3526.0	
	Lower limb	6	457.9	324.6	1588.0	
	Thorax	6	580.9	50.24	1780.0	
Tumour volume	Head and neck	27	39.25	6.28	205.2	0.5538
	Upper limb	5	47.1	22.5	71.44	
	Lower limb	5	157.0	34.02	2077	
	Thorax	6	30.09	5.36	202.5	
Specimen volume	Scalp	13	327.1	254.3	960.3	<0.0001 *
	Facial	4	269.5	50.5	642.	
	Ocular	32	19.89	6.67	60.45	
	Nasal	2	246.0	115.1	376.8	
	Cervical	3	1047	241.8	2638	
	Labial	1	3925	3925	3925	
Tumour volume	Scalp	10	94.2	30.88	361.1	0.0684 *
	Facial	4	196.8	50.63	239.7	
	Ocular	10	7.33	4.97	18.32	
	Nasal	2	348.5	131.9	565.2	
	Cervical	0	N/A			
	Labial	1	157	157	157	
Tumour volume	Benign	38	43.7	6.28	192.6	0.2546
	Malignant	3	131.9	62.8	34,540.0	
	Atypical	3	157.0	9.42	816.4	
Specimen volume	Benign	73	125.6	20.93	888.6	0.3845
	Malignant	2	22,038	115.1	43,960	
	Atypical	3	327.1	196.3	523.3	
Tumour volume	Sweat gland	33	54.43	7.33	291.8	0.5880
	Hair follicle	11	40.3	10.47	157.1	
Specimen volume	Sweat gland	62	113.6	18.84	612.3	0.1189
	Hair follicle	16	254.3	99.43	1645.0	

* Labial and nasal lesions were excluded from the analysis due to low frequencies, N/A—not assessed.

**Table 8:** Correlation of patient age and year of diagnosis with tumour and surgical specimen volumes.

Variables		*p*	*r*	95% CI
Age	Specimen volume	0.1319	−0.1721	−0.3858 to 0.0591
	Tumour volume	0.3280	−0.1510	−0.4360 to 0.1616
Year of diagnosis	Specimen volume	0.1445	−0.1668	−0.3811 to 0.06458
	Tumour volume	0.7295	+0.0536	−0.2557 to 0.3530

**Table 9:** Surgical resection margin analysis of adnexal tumours according to sex, topography and clinicopathological characteristics.

Surgical Resection		(<1 mm)		Free Margins		Infiltrated		Cannot Be Evaluated		*p*
		*n*	%	*n*	%	*n*	%	*n*	%	
Sex	Female	3	5.88	41	80.39	2	3.92	5	9.80	0.6992
	Male	1	3.23	27	87,10	2	6.45	1	3.23	
Topography	Head and neck	1	1.72	52	89.66	3	5.17	2	3.45	0.0069
	Upper limb	1	10.00	7	70.00	1	10.00	1	10.00	
	Lower limb	0	0.00	3	50.00	0	0.00	3	50.00	
	Thorax	2	28.57	5	71.43	0	0.00	0	0.00	
	Scalp	1	7.70	10	76.92	0	0.00	2	15.38	0.0720
	Facial	0	0.00	3	75.00	1	25.00	0	0.00	
	Ocular	0	0.00	34	97.14	1	2.86	0	0.00	
	Nasal	0	0.00	2	100.00	0	0.00	0	0.00	
	Cervical	0	0.00	2	66.66	1	33.33	0	0.00	
	Labial	0	0.00	1	100.00	0	0.00	0	0.00	
Lineage	Hair follicle	1	5.88	12	70.59	4	23.53	0	0.00	0.0031
	Sweat gland	3	3.62	56	86.15	0	0.00	6	9.23	
Behaviour	Benign	3	4.00	66	88.00	3	4.00	3	4.00	0.0003
	Malignant	1	25.00	2	50.00	1	25.00	0	0.00	
	Atypical	0	0.00	0	0.00	0	0.00	3	100.00	

**Table 10:** Tumour volume, surgical specimen volume, and patient age according to surgical margin status.

**Variables**		***n***	**Median (mm^3^)**	**Q1 (mm^3^)**	**Q3 (mm^3^)**	***p***
Tumour volume	(<1 mm)	3	47.1	2.62	34,540	0.5559
	Free margins	35	54.43	8.37	205.2	
	Infiltrated	2	22.5	4.71	40.3	
	Cannot be evaluated	4	162.2	46.32	654.2	
Specimen volume	(<1 mm)	4	668.8	170.0	33,234	0.0901
	Free margins	65	96.29	18.84	800.7	
	Infiltrated	3	403.0	241.8	1842	
	Cannot be evaluated	6	359.8	166.8	883.1	
**Variables**		***n***	**Mean (years)**		**SD (years)**	***p***
Age	(<1 mm)	4	52		±27	0.2294
	Free margins	68	60		±14	
	Infiltrated	4	38		±26	
	Cannot be evaluated	6	43		±21	

SD—standard deviation.

**Table 11:** Clinicopathological features of atypical and malignant tumours.

Case	Diagnosis	Age	Sex	Site	Main Histological Features	IHC/Ki-67	Follow-Up
1	Atypical spiradenoma	24	M	Scalp	Focal cytological atypia, focally increased mitotic activity; recurrent lesion with two relapses	p63+, CK7+; Ki-67 up to 15%	Tumour-free; under dermatologic surveillance for the past 3 years
2	Atypical spiradenoma	31	F	Lower limb	Focal atypical features, increased mitotic activity	p63+, CK7+; Ki-67 increased	Tumour-free 6-month follow-up; current data not available
3	Atypical spiradenoma	30	F	Lower limb	Focal atypical features, increased mitotic activity	p63+, CK7+; Ki-67 increased	No data available for follow-up
4	Eccrine porocarcinoma	40	F	Breast	Infiltrative growth, marked cytological atypia, focal necrosis, high mitotic activity	AE1/AE3+, CD117+, focal SOX10+; Ki-67 approx. 80%	Oncological surveillance for the past two years
5	Eccrine porocarcinoma	40	M	Scalp	Infiltrative growth, comedonecrosis, high mitotic count	CK7+, CD117+, Ki-67 over 50%	No recurrence, oncological surveillance for over a year
6	Basal cell carcinoma associated with trichoblastoma	62	M	Eyelid	Trichoblastoma component associated with basal cell carcinoma component; architectural irregularity, mitotic activity, increased Ki-67 in carcinoma component; three relapses until negative margins	Ki-67 increased in carcinoma component and low in the trichoblastoma component	Local radiation therapy was performed. The patient has been tumour-free for the past two years.
7	Trichilemmal carcinoma	66	M	Thorax	Solid lobulated growth, cytological atypia, prominent nucleoli, increased mitotic activity, surface keratinisation and infiltrative growth	Ki-67 increased approx. 40%	No data available for follow-up

## Data Availability

Data are available from the corresponding author upon reasonable request, subject to institutional restrictions.

## Abbreviations

The following abbreviations are used in this manuscript:

34βE12	High-molecular weight cytokeratin antibody (Clone 34βE12)
AE1/AE3	Pan-cytokeratin antibody cocktail AE1/AE3
ANOVA	Analysis of variance
AZ	Arizona
CATs	Cutaneous Adnexal Tumours
CD117	Cluster of differentiation 117 (c-KIT)
CI	Confidence interval
CK7	Cytokeratin 7
COVID-19	Coronavirus disease 2019
CTK	Cytokeratin
DAB	3,3′-Diaminobenzidine
EMA	Epithelial membrane antigen
ER	Estrogenic receptor
H&E	Haematoxylin&eosin
HMW	High-molecular weight
HPF	High-power field
IHC	Immunohistochemistry
IQR	Interquartile range
Ki-67	Ki-67 proliferation index
MSO	Microsoft office
NOS	Not otherwise specified
PRAME	Preferentially expressed antigen in melanoma
Q1	First quartile
Q3	Third quartile
Q-Q Plot	Quantile–quantile plot
SD	Standard deviation
SOX10	SRY-box transcription factor 10
USA	United States of America
WHO	World Health Organization

## Appendix A. Immunohistochemistry

A complete list of the antibody clones used in this study, together with their corresponding specifications, is provided in the table below.

CTK HMW (34βE12)	CONFIRM Anti-Keratin (34βE12) Mouse Monoclonal Primary Antibody, VENTANA
p63 (4A4)	Anti-p63 (4A4), Mouse Monoclonal Primary Antibody, VENTANA
Cytokeratin 7 (SP52)	CONFIRM Anti-Cytokeratin 7 (SP52), Rabbit Monoclonal Antibody, VENTANA
EMA (E29)	CONFIRM Anti-EMA (E29), Mouse Monoclonal Antibody, VENTANA
CTK AE1/AE3 (AE1/AE3/PCK26)	Anti-Pan Keratin (AE1/AE3/PCK26) Primary Antibody, VENTANA
CD 117 (9.7)	PATHWAY Anti-c-KIT (9.7) Primary Antibody, VENTANA
SOX-10 (SP267)	SOX-10(SP267) Mouse Monoclonal Primary Antibody, CELL MARQUE
ESTROGEN REC (ER) (SP1)	CONFIRM Anti-Estrogen Receptor (ER) (SP1) Rabbit Monoclonal Primary Antibody, VENTANA
GATA 3 (L50-823)	GATA3 (L50-823) Mouse Monoclonal Primary Antibody, VENTANA
Ki-67 (30-9)	Ki-67 (30-9) Rabbit Monoclonal Primary Antibody, VENTANA
